# 2,4-Dimethoxy-6-Methylbenzene-1,3-diol, a Benzenoid From *Antrodia cinnamomea*, Mitigates Psoriasiform Inflammation by Suppressing MAPK/NF-κB Phosphorylation and GDAP1L1/Drp1 Translocation

**DOI:** 10.3389/fimmu.2021.664425

**Published:** 2021-05-14

**Authors:** Shih-Yi Chuang, Chi-Yuan Chen, Shih-Chun Yang, Ahmed Alalaiwe, Chih-Hung Lin, Jia-You Fang

**Affiliations:** ^1^ Pharmaceutics Laboratory, Graduate Institute of Natural Products, Chang Gung University, Taoyuan, Taiwan; ^2^ Graduate Institute of Health Industry Technology, Chang Gung University of Science and Technology, Taoyuan, Taiwan; ^3^ Research Center for Food and Cosmetic Safety and Research Center for Chinese Herbal Medicine, Chang Gung University of Science and Technology, Taoyuan, Taiwan; ^4^ Tissue Bank, Chang Gung Memorial Hospital, Taoyuan, Taiwan; ^5^ Department of Cosmetic Science, Providence University, Taichung, Taiwan; ^6^ Department of Pharmaceutics, College of Pharmacy, Prince Sattam Bin Abdulaziz University, Al Kharj, Saudi Arabia; ^7^ Center for General Education, Chang Gung University of Science and Technology, Taoyuan, Taiwan; ^8^ Department of Anesthesiology, Chang Gung Memorial Hospital, Taoyuan, Taiwan

**Keywords:** *Antrodia cinnamomea*, 2,4-dimethoxy-6-methylbenzene-1,3-diol, psoriasis, macrophage, GDAP1L1, Drp1

## Abstract

*Antrodia cinnamomea* exhibits anti-inflammatory, antioxidant, and immunomodulatory activities. We aimed to explore the antipsoriatic potential of 2,4-dimethoxy-6-methylbenzene-1,3-diol (DMD) derived from *A. cinnamomea*. The macrophages activated by imiquimod (IMQ) were used as the cell model for examining the anti-inflammatory effect of DMD *in vitro*. A significantly high inhibition of IL-23 and IL-6 by DMD was observed in THP-1 macrophages and bone marrow-derived mouse macrophages. The conditioned medium of DMD-treated macrophages could reduce neutrophil migration and keratinocyte overproliferation. DMD could downregulate cytokine/chemokine by suppressing the phosphorylation of mitogen-activated protein kinases (MAPKs) and NF-κB. We also observed inhibition of GDAP1L1/Drp1 translocation from the cytoplasm to mitochondria by DMD intervention. Thus, mitochondrial fission could be a novel target for treating psoriatic inflammation. A psoriasiform mouse model treated by IMQ showed reduced scaling, erythema, and skin thickening after topical application of DMD. Compared to the IMQ stimulation only, the active compound decreased epidermal thickness by about 2-fold. DMD diminished the number of infiltrating macrophages and neutrophils and their related cytokine/chemokine production in the lesional skin. Immunostaining of the IMQ-treated skin demonstrated the inhibition of GDAP1LI and phosphorylated Drp1 by DMD. The present study provides insight regarding the potential use of DMD as an effective treatment modality for psoriatic inflammation.

## Introduction

Psoriasis is one of the most common autoimmune skin disorders. Patients with psoriasis are characterized by red and thick plaques covered with silver multilayered scales. IL-23/helper T cell type 17 (Th17) axis is recognized to have a key role in psoriasis (1). Typical histopathology of psoriatic lesions includes epidermal hyperplasia, elongated rete ridge, and immune cell infiltration. The estimated global prevalence of psoriasis is 2%−3% (2). Approximately 80% of the patients with psoriasis are classified as mild-to-moderate (3), for which topical drug treatment is feasible. However, the topical treatment is far from satisfactory due to the time-consuming therapeutic course, frustration with efficacy, and side effects (4). There is an urgent need to develop new antipsoriatic agents with improved therapeutic efficiency and safety. The development of antipsoriatic candidates from natural resources can potentially achieve the purposes of superior therapeutic effectiveness and fewer adverse effects (5). Nearly 39%−62% patient population in Asia and the Middle East use complementary and alternative medicine for treating psoriasis (6) And nearly 47% of the patients in South Europe use plant extracts as a remedy against psoriasis (7).

Many natural compounds derived from mushrooms are known to exhibit anti-inflammatory and immunomodulatory activities (8). The mushroom *Antrodia cinnamomea* is used as a medicinal herb because of its biological properties. *A. cinnamomea* is traditionally used to treat diarrhea, abdominal pain, hypertension, cancers, and itchy skin (9). The extracts and bioactive compounds from *A. cinnamomea* are reported to show biological effects such as anti-inflammatory, antioxidant, antitumor, antihyperlipidemic, and hepatoprotective activities (10). The ethanolic extract derived from *A. cinnamomea* inhibits Th17 cell infiltration in the dermis of the psoriasiform lesion and can thus be a therapeutic option for psoriasis (11). Previous investigations (12, 13) suggest the anti-inflammatory activity of some benzenoids isolated from *A. cinnamomea* on the activated T cells and macrophages. Likewise, we demonstrated in a previous study (14) that the benzenoid 2,4-dimethoxy-6-methylbenzene-1,3-diol (DMD) from *A. cinnamomea* exerts anti-inflammatory activity in atopic dermatitis-like skin in mice. In that study, we mainly explored the therapeutic potential of DMD on psoriasis treatment based upon the cell-based and *in vivo* animal studies.

Psoriasis is generally regarded as a T cell-mediated disease. Nevertheless, there is increasing evidence indicating that macrophages also play an essential role in psoriasis pathogenesis (15). Macrophages differentiate from monocytes in the blood, enter the host tissue, and are influenced by the local environment. Macrophages are largely infiltrated in the dermal layer of psoriasis to release cytokines IL-23, IL-6, and TNF-α during the development of the lesion (16). We aimed to explore an effective strategy to treat psoriasis by regulating macrophage activation and to elucidate the possible mechanisms of DMD-mediated inhibition of inflammation using the macrophages (the differentiated THP-1 cells) as the cell model. Imiquimod (IMQ) is a Toll-like receptor (TLR)7 ligand which acts as an immune stimulator for macrophages (17). We used IMQ to activate macrophages and induce psoriasis-like plaque in mice for evaluating the anti-inflammatory effect of DMD in psoriasis treatment.

## Materials And Methods

### Reagents and Antibodies

Menadione, tert-butylhydroquinone (TBHQ) and phorbol 12-myristate 13-acetate (PMA) were purchased from Sigma-Aldrich (St. Louis, MO, USA). IMQ cream (Aldara^®^, 5%) was acquired from 3M Pharmaceuticals (Leicestershire, UK). Phospho (p)-JNK, p-ERK, p-p38, p-p65, JNK, ERK, p38, CCR7, Drp1 and GAPDH antibodies were purchased from Santa Cruz Biotechnology (Santa Cruz, CA, USA). F4/80 and Ly6G antibodies were purchased from Abcam (Cambridge, MA, USA). The anti-Ly6G antibody was purchased from eBiosciences (San Diego, CA, USA). GDAP1L1 and mtHSP70 antibodies were purchased from Invitrogen (Carlsbad, CA, USA). The antibody targeting phospho-Drp1-S616 was obtained from Biorbyt (St. Louis, MO, USA).

### DMD From *A. cinnamomea*

DMD was obtained by partitioning and silica gel column chromatography. The detailed information of the extraction and isolation was described earlier (14). The chemical structure of DMD is illustrated in **Supplementary Figure 1**.

### Cell Lines, Primary Cells, and Cell Culture

Human monocytic leukemia THP-1 cell line was maintained in RPMI 1640 supplemented with 10% heat-inactivated FBS and 100 U/mL penicillin and streptomycin. Before the experiments, THP-1 cells were differentiated into macrophages by treating with phorbol 12-myristate 13-acetate (100 ng/mL) for 36 h, followed by overnight incubation in a fresh medium. Bone marrow was collected from the femur and tibia of BALB/c mice to generate bone marrow-derived macrophages (BMDMs) following an earlier published protocol (18). For testing the effect of DMD on activated THP-1 and BMDMs, the cells were pretreated with DMD for 1 h and then incubated with IMQ (10 μg/mL) for 24 h. HaCaT cells, the immortalized cell line of human keratinocytes, were cultured in DMEMs supplemented with 10% FBS and 100 U/mL penicillin-streptomycin at 37°C. Human primary neutrophils were obtained from healthy, 20-30 years old volunteers using a protocol approved by the Institutional Review Board at Chang Gung Memorial Hospital (201701925B0). All volunteers provided written informed consent for participation. The neutrophils were purified by sedimentation prior to centrifugation and erythrocyte lysis following the protocol in a previous report (19).

### Cytotoxicity Assay

The cytotoxicity of DMD was studied by 3-(4,5-dimethylthiazol-2-yl)-2,5-diphenyltetrazolium bromide (MTT) analysis. The macrophages were cultured in DMEM at a density of 2 x 10^6^ cells/well and incubated at 37°C for 24 h. Then DMD (1−40 μg/mL) was added into the cell suspension and incubated for 24 h. The cells in a blank medium were used as the control. MTT (0.5 mg/mL) present in culture medium (200 μL) was incorporated in the cell suspension, which was further incubated at 37°C for 4 h. The THP-1 viability was detected by a spectrophotometer at 570 nm. The trypan blue assay was also used to evaluate the cell viability. After treating DMD for 24 h, the cells were removed and stained with 0.4% trypan blue. The number of unstained viable cells was counted in a hemocytometer under light microscope (Leica DMi8).

### Total RNA Extraction and Real-Time Quantitative Polymerase Chain Reaction (RT-qPCR)

Total cellular RNA was extracted using the Direct-zol kit with RNase-free DNase 1 digestion to remove genomic DNA contamination according to the manufacturer’s instructions. Reverse transcription to cDNA was performed by iScript cDNA Synthesis kit. RT-qPCR was carried out by a CFX Connect RT PCR Detection System using iQ SYBR Green Supermix. The level of GAPDH was used to normalize the mRNA level. The primer sequences used for amplification from mouse and human species are listed in **Supplementary Table 1** and **Supplementary Table 2**, respectively.

### Enzyme-Linked Immunosorbent Assay (ELISA)

The level of cytokines and chemokines in the supernatant of the cell medium was quantified using ELISA kits (BioLegend) according to the manufacturer’s instructions. The absorbance was measured at 450 nm on a microplate spectrophotometer. The concentration of cytokines and chemokines was estimated based on the corresponding standard curves.

### Immunoblotting

The level of mitogen-activated protein kinases (MAPKs), NF-κB, ganglioside-induced differentiation-associated protein 1 like 1 (GDAP1L1), dynamin-related protein 1 (Drp1), and Drp1 S616 were estimated by western blotting. The cells were collected and added to the lysis buffer. The nuclear pellets were obtained after centrifugation at 400 x *g* and 4°C for 5 min. After probe sonication, the protein fraction was obtained by centrifugation at 8,000 x *g* and 4°C for 10 min. For quantification, protein assay dye was mixed with the protein fraction, and separated by 10% acrylamide SDS-PAGE, and transferred to a polyvinylidene difluoride membrane. The membrane was incubated with the primary antibody (1:1000 dilution) at 4°C overnight. Subsequently, the membrane was washed using tris-buffered saline and incubated with horseradish peroxidase-conjugated secondary antibody (1:5000 dilution) for 1 h. The immunoreactive bands were detected by Western Lightning Plus-ECL. Anti-GAPDH or anti-actin antibody was used as the loading control.

### Wound Healing Assay

Fresh neutrophils (4 x 10^5^ cells/well) were seeded in a six-well plate and cultured in the complete DMEM medium. The cells were scraped using a 200 μL pipette tip to achieve a noncellular region, and the culture medium was then replaced by the conditioned medium of THP-1 cells after IMQ stimulation with or without DMD intervention (10 μg/mL). After 4 h, the migration number of the neutrophils was measured by using ImageJ software.

### Chemotaxis Assay

The neutrophil migration initiated by the conditioned medium of THP-1 cells was evaluated by the Boyden chamber migration analysis. Briefly, the isolated human neutrophils were added to DMEM supplemented with 0.25% BSA. The neutrophils (4 x 10^5^ cells/well) were added to the upper well of the Boyden chamber. The conditioned medium harvested from macrophages was added into the lower well. The plate was stored at 37°C for 4 h before placing it on ice, and 100 μL of 0.5 M EDTA was incorporated into the well at 4°C for 10 min. The well insert was removed and the cell suspension was collected. Then neutrophil count was estimated using a Moxi Z Mini-Automated Cell Counter kit.

### Isolation of Cytosolic and Mitochondrial Fractions

GDAP1L1 and Drp1 levels in cytosol and mitochondria of macrophages were determined by western blotting. The separation of mitochondria from cytoplasm was performed as described previously (18). In brief, the macrophages were collected, then the Mitochondria Isolation kit was used to separate the cellular components according to the manufacturer’s instructions. Cytoplasmic and mitochondrial proteins were quantified by western blotting using the same method described in the section of immunoblotting. The p38 and mitochondrial HSP70 (mtHSP70) were employed as the loading control for cytosolic and mitochondrial fractions, respectively.

### Immunofluorescence Staining

To appraise the expression of GDAP1L1 and Drp1 S616 in THP-1 cells, immunofluorescence staining was carried out. The cells were cultured on coverslips and then labeled with MitoTracker Red for staining mitochondria. The macrophages were fixed with 4% paraformaldehyde for 15 min at room temperature. The fixed macrophages were permeabilized with Triton X-100 for 5 min and then blocked with 1% BSA for 30 min. For immunofluorescence staining, primary antibodies against GDAP1L1 or Drp1 S616 were used followed by the incubation of secondary antibody conjugated with Alexa Fluor 488 or 594. The cells were monitored under a confocal microscope (Zeiss LSM780). Quantification and measurement of mitochondrial length was performed with MetaMorph (Molecular Devices) by an investigator blinded to experimental groups. Mitochondrial length was classified into three categories; <0.5 μm, 0.5−2 μm, and >2 μm. The resulting data were visualized using GraphPad Prism software.

### Immunoprecipitation

Total mitochondrial fraction extract (100 μg) was incubated with GDAP1L1 antibody overnight at 4°C. Protein A beads were added to the mixtures and incubated for 1 h at 4°C. After five washings with lysis buffer, the immunoprecipitants were subjected to SDS/PAGE and analyzed by immunoblotting as indicated antibodies.

### Animals

Female BALB/c mice (8-weeks old) were purchased from the National Laboratory Animal Center (Taipei, Taiwan). All animal experiments were approved by the Institutional Animal Care and Use Committee of Chang Gung University and complied with Directive 86/109/EEC from the European Commission (CGU108-101).

### IMQ-Induced Psoriasiform Skin in Mice

A psoriasis-like lesion was evoked on the back of the BALB/c mouse following a protocol from van der Fits et al., with modifications (20). The mouse received a daily topical dose of 62.5 mg 5% IMQ cream (Aldara, 3M) on the shaved area of the dorsal skin for five consecutive days. Before 30 min of IMQ cream treatment, 100 μL DMD (1 mg/mL) in PEG400/PBS (3:7) was topically administered on the back. The vehicle was dried during this 30-min period. The skin surface appearance was visualized using a portable digital magnifier (Mini Scope-V, M&T Optics). The subjective evaluation of the severity was estimated by the cumulative score (scaling plus erythema) with a scale from 0 to 8 based on the Psoriasis Area and Severity Index (PASI). Transepidermal water loss (TEWL) was detected by a Tewameter TM300 (Courage and Khazaka). The animals were sacrificed on day 6 for further histological examination and detection of cytokine/chemokine expression. The skin was extracted following a previously described method (21). The skin extract was used to determine proinflammatory mediators by RT-qPCR. The protocol for RT-qPCR was the same as that mentioned in the section for the *in vitro* THP-1 study.

### Histology

The skin sample was fixed in 10% formaldehyde and embedded in paraffin and then stained with hematoxylin and eosin (H&E). The unstained slice of skin sample was prepared for immunohistochemistry (IHC). After dewaxing and rehydration, the skin section was subjected to heat-induced epitope retrieval using the Bond Epitope Retrieval Solution 2 according to the manufacturer’s instructions, followed by the blocking with diluted normal serum. The section was incubated with rabbit polyclonal anti-mouse Ki67, Ly6G, F4/80, GDAP1L1, or Drp1 S616 antibody (1:100 dilution) for 1 h at room temperature, washed with 0.5% Tween 20 in saline, and subsequently incubated with biotinylated donkey anti-rabbit IgG at ambient temperature for 20 min. The photomicrographs were observed under an optical microscope (Leica DMi8). Quantification of the IHC-stained sections was performed by AlphaView software. Each section was examined independently by two investigators in a blinded manner. Numbers of positive cells were evaluated by counting the numbers of cells (original manifestation x200) for three sections of three mice per group.

### Statistical Analysis

The statistical differences in the data of different treatment groups were measured using the one-way analysis of variance followed by Tukey’s multiple comparison test. The data distribution was checked by Kolmogorov-Smirnov test. The levels of probability including 0.05, 0.01, and 0.001 were considered statistically significant.

## Results

### DMD Inhibits Cytokine/Chemokine Expression in Activated Macrophages

**Figure 1:**
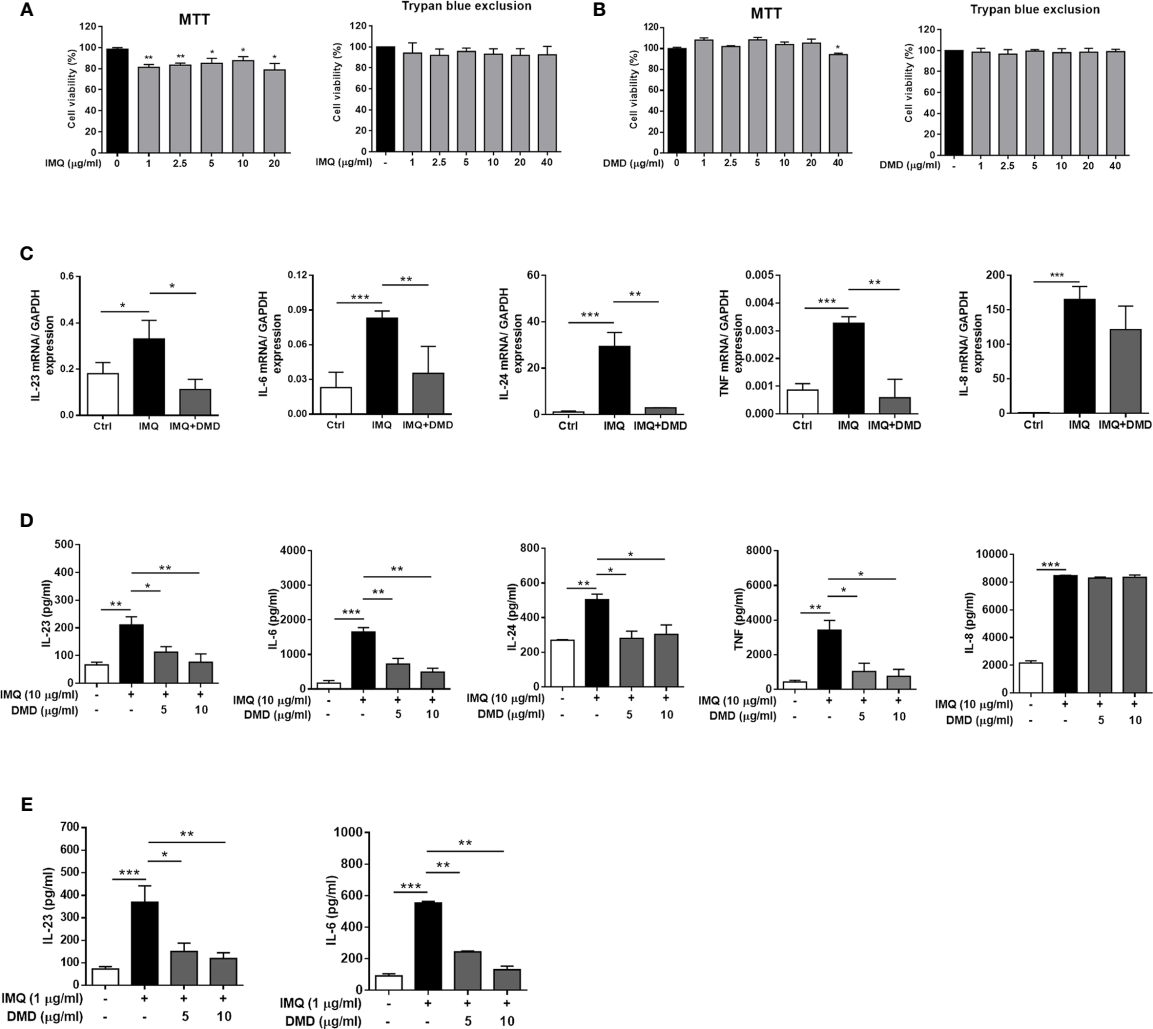
DMD inhibit IMQ-induced inflammatory cytokines in THP-1 and BMDMs. **(A, B)** Cell viability in THP-1 cells determined by MTT and trypan blue assays after incubation with IMQ and DMD in the indicated concentration, respectively, for 24 h (*n*=3). **(C)** RT-qPCR analysis of the cytokines in IMQ-stimulated THP-1 cells following DMD treatment (*n*=3). **(D)** ELISA analysis of the cytokines in IMQ-stimulated THP-1 cells following DMD treatment (*n*=3). **(E)** ELISA analysis of the cytokines in IMQ-stimulated BMDMs following DMD treatment (*n*=3). The data shown are representative of three independent experiments. GAPDH is served as an internal control. *P < 0.05, **P < 0.01, and ***P < 0.001 when compared to IMQ group. Data are represented as mean ± SEM.

IMQ was used as the activator to evoke the macrophage stimulation in this study. We tested the effect of IMQ on THP-1 viability to determine its cytotoxicity to the macrophages. Although slight cytotoxicity determined by MTT assay was found after IMQ treatment, the viability could still surpass 80% for the activator concentrations between 1 and 20 μg/mL (the left panel of **Figure 1A**). In addition to the evaluation of cell viability, MTT assay is a method to assess cell metabolic activity. The MTT assay is dependent on the mitochondrial respiration. The slight reduction of MTT by IMQ may suggest the involvement of IMQ in the metabolic activity of THP-1 cells. No cell viability reduction by IMQ was detected using trypan blue analysis (the right panel of **Figure 1A**), demonstrating a minimal cytotoxicity of IMQ. Then, an IMQ dose of 10 μg/mL was used in the subsequent experiments. Up to a concentration of 20 μg/mL, DMD had no effect on the viability for both MTT and trypan blue analyses (**Figure 1B**), while following the treatment with 40 μg/mL DMD the viability percentage was 94% in the MTT assay. The non-cytotoxic dose of 10 μg/mL DMD was selected to investigate the anti-inflammatory effect on macrophages.

**Figure 2:**
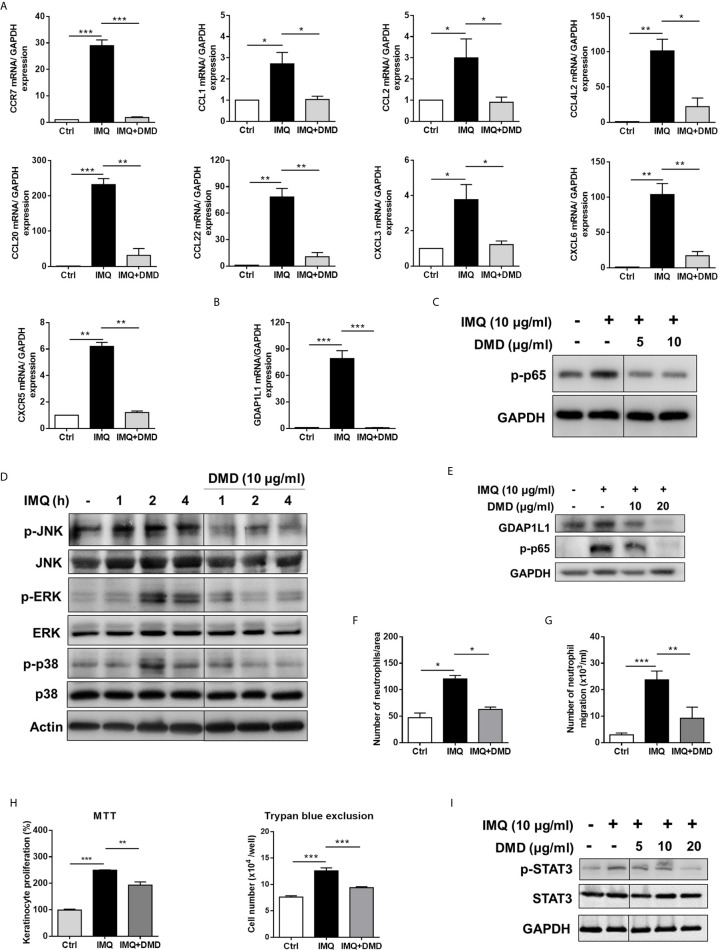
DMD inhibit IMQ-induced activation in THP-1 cells through MAPK and NF-κB pathways, and block neutrophil invasion and keratinocyte proliferation. **(A)** RT-qPCR analysis of the chemokines in IMQ-stimulated THP-1 cells following DMD treatment (*n*=4). **(B)** RT-qPCR analysis of GDAP1L1 in IMQ-stimulated THP-1 cells following DMD treatment (*n*=4). **(C–E)** THP-1 cells were treated with IMQ in the presence or absence of DMD. Immunoblotting analysis of phosphorylation of NF-κB, MAPKs, and GDAP1L1 was determined, respectively. **(F)** Neutrophil migration was measured by wound healing assay. Neutrophils were treated with the conditioned medium of THP-1 cells for 24 h to determine the rate of migration into the scratched area. Wound healing was quantified by measuring the distance between scratch edges at 0 and 24 h. **(G)** Neutrophil invasion was measured by the Boyden chamber invasion assay. Neutrophils were loaded on the top chambers. The cells were allowed to invade for 4 h, and the rate of invasion was quantified (*n*=4). **(H)** Keratinocyte proliferation induced by the conditioned medium of THP-1 cells was measured using the MTT and trypan blue assays (*n*=3). **(I)** Immunoblotting of the phosphorylation of STAT3 in keratinocytes stimulated with the conditioned medium of THP-1 cells for 24 h. One out of three independent experiments is shown. *P < 0.05, **P < 0.01, and ***P < 0.001 when compared to IMQ group. Data are represented as mean ± SEM.

To further elucidate the role of DMD on the cytokine expression, RT-qPCR analysis was carried out for the five genes of IL-23, IL-6, IL-24, TNF, and IL-8. IMQ was able to increase the expression of these inflammatory factors with a statistical significance (**Figure 1C**). The mRNA detection verified a decrease in cytokine level after DMD versus IMQ stimulation. At 10 μg/mL, DMD totally inhibited IL-23, IL-6, IL-24, and TNF to the baseline control. The expression of the cytokines in macrophages was further confirmed by ELISA. The protein and mRNA expression showed a consistent trend (**Figure 1D**). BMDMs, the primary macrophages derived from the mouse, were used as another macrophage model for treating the effect of DMD on inflammation attenuation. Similar to THP-1 cells, IMQ induced some cytotoxicity against BMDMs (**Supplementary Figure 2**). DMD produced a limited cytotoxicity on BMDMs. The viability of BMDMs could be maintained to 75% after DMD treatment at the highest concentration (40 μg/mL). *In vitro*, BMDMs also expressed IL-23 and IL-6 at high levels after IMQ stimulation (**Figure 1E**), and this upregulation could be reduced in the DMD-treated groups. To understand the capacity of chemokines, we analyzed mRNA expression of nine chemokines in IMQ-activated macrophages and observed a high level of CCR7, CCL1, CCL2, CCL4L2, CCL20, CCL22, CXCL3, CXCL6, and CXCR5 in stimulated THP-1 (**Figure 2A**), and this could be abolished by DMD treatment. This result suggests the involvement of DMD in the regulation of macrophage chemotaxis. We also observed upregulated GDAP1L1 mRNA by IMQ and a 70-fold decrease to that of the baseline after DMD treatment (**Figure 2B**). GDAP1L1 is a paralog of GDAP1 and predominantly governs mitochondrial dynamics.

### DMD Inhibits Cytokine/Chemokine Expression by Blocking the MAPK/NF-κB Signaling

To elucidate the signaling pathways through which DMD attenuated the inflammation, we checked the possible role of NF-κB. Western blot analysis displayed that IMQ upregulated the expression of p-p65 that was a subunit of p-NF-κB (**Figure 2C**), while a significantly opposite effect was observed after DMD treatment in macrophages. DMD inhibited the phosphorylation of p65 by 2-fold as measured by densitometry (**Supplementary Figure 3A**). To examine the possible role of MAPKs in the inflammation suppression by DMD, we studied the IMQ-induced phosphorylation of JNK, ERK, and p38 in macrophages. The maximum expression of the phosphorylated MAPKs was detected at 2 h after IMQ stimulation (**Figure 2D** and **Supplementary Figure 3B**), while DMD could reverse the phosphorylation, clearly indicating the anti-inflammatory activity of DMD through the downregulation of MAPKs and NF-κB. GDAP1L1 is an important marker that showed a significant decrease after DMD treatment of activated THP-1 macrophages. Immunoblotting revealed that GDAP1L1 protein level increased after IMQ and arrested by DMD treatments in a dose-dependent manner (**Figure 2E** and **Supplementary Figure 3C**).

### The Conditioned Medium of DMD-Treated Macrophages Prevents Neutrophil Migration and Keratinocyte Proliferation

In psoriasis, macrophage chemotaxis may mediate the interplay between the macrophages and the other cells. To gain an in-depth understanding of the chemotaxis, we validated the effect of IMQ-activated macrophage conditioned medium on neutrophil invasion and keratinocyte proliferation. The wound-healing assay showed that DMD-treated macrophage medium suppressed wound closure in neutrophils (**Figure 2F**). Moreover, the DMD-treated group had lowered the average number of migrating neutrophils permeating the transwell membrane (**Figure 2G**). The conditioned medium from IMQ-stimulated macrophages promoted keratinocyte proliferation by about 2-fold as measured by both MTT and trypan blue assays (**Figure 2H**). This effect was inhibited by DMD at 10 μg/mL. The keratinocyte proliferation in psoriasis is in close association with the signal transducer and activator of transcription 3 (STAT3) pathway. The IMQ-treated conditioned medium enhanced STAT3 activation in keratinocytes, which was observed in the form of increased p-STAT3 (**Figure 2I** and **Supplementary Figure 3D**), which was considerably reduced upon the intervention of DMD. Our data demonstrated that DMD was effective in suppressing neutrophil and keratinocyte activation *via* the inhibition of macrophage chemotaxis.

### GDAP1L1/Drp1 Axis Participates in DMD-Induced Inhibition on Macrophage Activation

**Figure 3:**
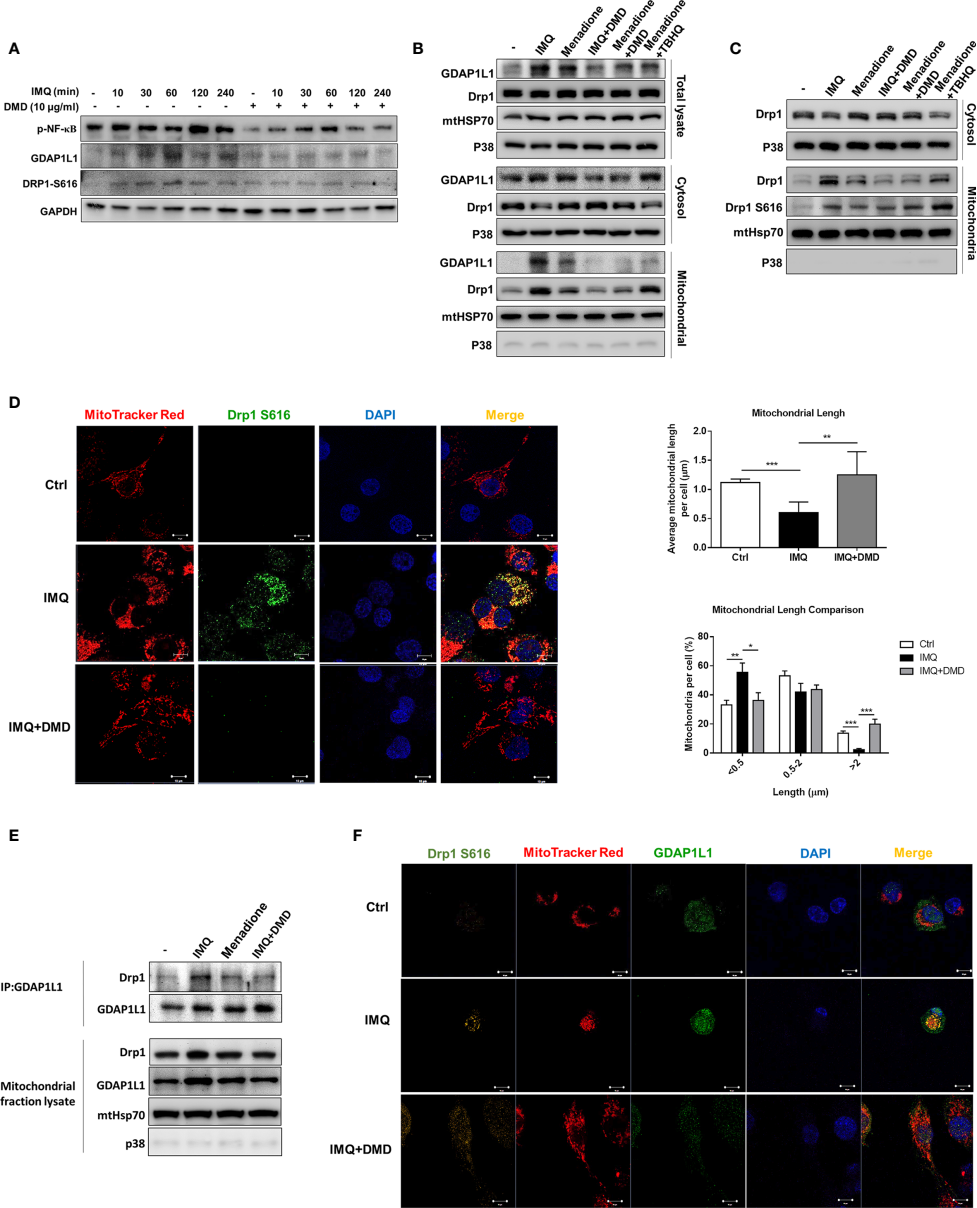
DMD inhibit IMQ-induced activation in THP-1 cells through decreasing Drp1 phosphorylation and GDAP1L1/Drp1 translocation. **(A)** Immunoblotting of GDAP1L1 and Drp1 S616 in IMQ-stimulated THP-1 cells following DMD treatment. **(B)** THP-1 cells were pretreated by DMD or TBHQ and then stimulated with IMQ or menadione for 2 h. Cells were fractionated into cytosolic and mitochondrial fractions and subjected to immunoblotting assay to determine GDAP1L1 and Drp1. **(C)** THP-1 cells were pretreated by DMD or TBHQ and then stimulated with IMQ or menadione for 2 h. Cells were fractionated into cytosolic and mitochondrial fractions and subjected to immunoblotting assay to determine Drp1 and its phosphorylated form (Drp1 S616). **(D)** THP-1 cells were stimulated with IMQ for 2 h, and then stained with MitoTracker Red. Cells were fixed and immunostained with the antibody against Drp1 S616 followed by confocal microscopy. Average mitochondrial length (μm) and comparison of mitochondrial length distribution in the indicated groups is shown in the right panel. **(E)** Immunoprecipitation of GDAP1L1 and Drp1 in mitochondria of THP-1 cells as shown in the immunoblotting. **(F)** THP-1 cells were stimulated with IMQ for 2 h, and then stained with MitoTracker Red. Cells were fixed and immunostained with the antibody against GDAP1L1 or Drp1 S616 followed by confocal microscopy. Scale bars, 10 μm. All experiments were repeated two or three times with similar results.

According to the cell-based study, we found a possible role of GDAP1L1 in the inhibition of activated macrophage by DMD. GDAP1L1 is a mitochondrial fission factor. Mitochondrial fission and fusion are required for maintaining mitochondrial functions related to biogenesis, apoptosis, neurodegeneration, and inflammation. Following IMQ stimulation, the GDAP1L1 level was significantly increased at the indicated times as verified by densitometry (**Figure 3A** and **Supplementary Figure 4**). DMD treatment at 10 μg/mL blocked IMQ-stimulated GDAP1L1 expression. The activity of GDAP1L1 to induce mitochondrial fission depends upon another fission factor Drp1. The fission needs the phosphorylated Drp1 at the S616 site and translocation of Drp1 from the cytoplasm to mitochondria. We found an increase in IMQ-mediated Drp1 phosphorylation in THP-1 (**Figure 3A**), and this elevation could be reduced by DMD. GDAP1L1 is a cytosolic protein that is also delivered to the mitochondria in response to external stimuli. We found the association between GDAP1L1 and mitochondria after IMQ treatment (**Supplementary Figure 5**), which increased following the increase in treatment duration. To appraise whether DMD affected GDAP1L1 and Drp1 location, cytosol and mitochondria were isolated from IMQ-treated macrophages and more GDAP1L1 was expressed in the mitochondria after IMQ stimulation (**Figure 3B**). We found a minimal expression of the cytosolic specific marker p38 in the mitochondrial fraction. Menadione is a quinone-related derivative used as a positive control in oxidative stress-induction studies. Mitochondrial translocation of GDAP1L1 was found after menadione treatment. DMD not only reduced GDAP1L1 production but also decreased IMQ- and menadione-induced GDAP1L1 translocation. Menadione is conjugated to form menadione-S-glutathione by glutathione S-transferase to induce stress. Glutathione conjugation detoxifies tertiary butylhydroquinone (TBHQ). TBHQ could reduce the GDAP1L1 transfer to mitochondria sensitized by menadione. IMQ and menadione also strongly increased the expression of Drp1 in mitochondria without a significant change of total Drp1. DMD treatment abolished the elevated GDAP1L1 in mitochondria. However, this phenomenon was not detected in the case of menadione-treated cells intervened by TBHQ.

We then examined the phosphorylated Drp1 at S616 in mitochondria. The level of mitochondrial Drp1 and Drp1 S616 increased in THP-1 stimulated with IMQ and menadione (**Figure 3C**) and this increase in the active form of Drp1 in mitochondria was blocked by DMD, as was confirmed by immunofluorescence analysis (the left panel of **Figure 3D**). Colocalization of Drp1 S616 with mitochondria was observed using double immunofluorescence staining. The estimation of mitochondrial length showed a decreased average length after IMQ intervention (the upper right panel of **Figure 3D**), suggesting a fission of mitochondria after an inflammation stimulation. DMD treatment could reverse this reduction to the control baseline level. We also found an increased proportion of mitochondria with short length (<0.5 μm) by IMQ stimulation (the lower right panel of **Figure 3D**). Again, this increase could be reversed to baseline control by DMD. Based on the above-mentioned results, we speculated that Drp1 might complex with GDAP1L1. We employed immunoprecipitation to check if GDAP1L1 and Drp1 were associated to interact with each other. The GDAP1L1 antibody could successfully pull down both GDAP1L1 and Drp1 (**Figure 3E**). GDAP1L1 indeed coprecipitated with Drp1 when transiently expressed in THP-1 cells. Both Drp1 S616 and GDAP1L1 expression was upregulated in mitochondria after IMQ stimulation, as shown by the immunofluorescence image (**Figure 3F**). An appreciable overlap was observed between both fission factors and mitochondria. GDAP1L1 possibly colocalized with phosphorylated Drp1 in mitochondria. We verified that the DMD-induced inflammation suppression was mediated by regulating GDAP1L1/Drp1 translocation to mitochondria.

### DMD Alleviates Psoriasiform Lesion in IMQ-Induced Mouse Model

**Figure 4:**
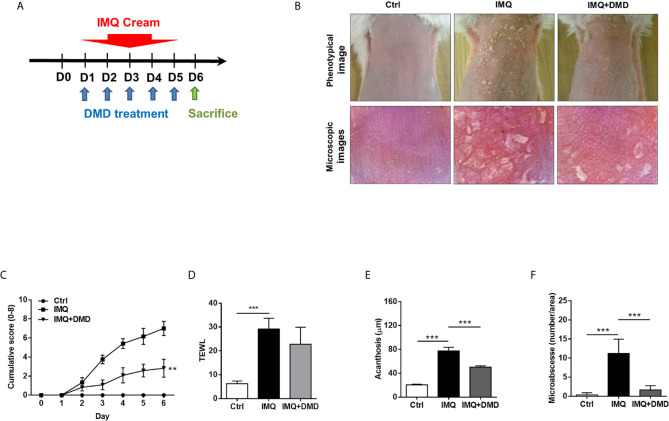
DMD attenuate IMQ-induced psoriasis-like inflammation in a mouse model. **(A)** Scheme of the experimental protocol for AC or DMD treatment in IMQ-induced psoriasis-like inflammation model in mice. **(B)** Phenotypical and microscopic images of IMQ-induced psoriasis-like inflammation on mouse skin with and without the treatment by AC or DMD after 5 days. **(C)** The cumulative score (scaling plus erythema from 0 to 4 each) is depicted. **(D)** TEWL was measured on Day 6. **(E)** Epidermal thicknesses measured according to H&E-stained histology. **(F)** Munro’s microabscesses measured using an image analysis system. All experiments were performed at least three times. **P < 0.01 and ***P < 0.001 when compared to IMQ group. Scale bar, 100 μm. Data are represented as mean ± SEM. (*n*=6).

IMQ was topically applied on mouse skin to generate psoriasis-like plaque for evaluating the anti-inflammatory activity of DMD. The pure compound from *A. cinnamomea* was topically administered on the skin of the back treated with IMQ cream for five consecutive days (**Figure 4A**). Compared to the healthy skin, scaling, erythema, and thickening in the IMQ-treated skin were observed in the macroscopic and microscopic visualization of the skin surface (**Figure 4B**). These symptoms suggest a typical psoriasis feature. DMD-treated groups displayed the clearance of these signs compared to the only IMQ-treated group. The cumulative score estimated by scaling and redness was significantly reduced by DMD as compared to the only IMQ treatment group (**Figure 4C**). TEWL, as an indicator of cutaneous barrier property, exhibited a 4-fold increase after IMQ stimulation (**Figure 4D**). No improvement of skin barrier dysfunction was detected after DMD intervention. The epidermal thickness increased by about 3-fold by IMQ activation as compared to normal skin (**Figure 4E**). This histological sign was relieved by DMD. The epidermal thickness of IMQ-treated skin was reduced from 77 to 50 μm after DMD treatment. The Munro’s microabscess in IMQ-stimulated lesion was reduced by 6.8-fold after treatment of DMD (**Figure 4F**).

**Figure 5:**
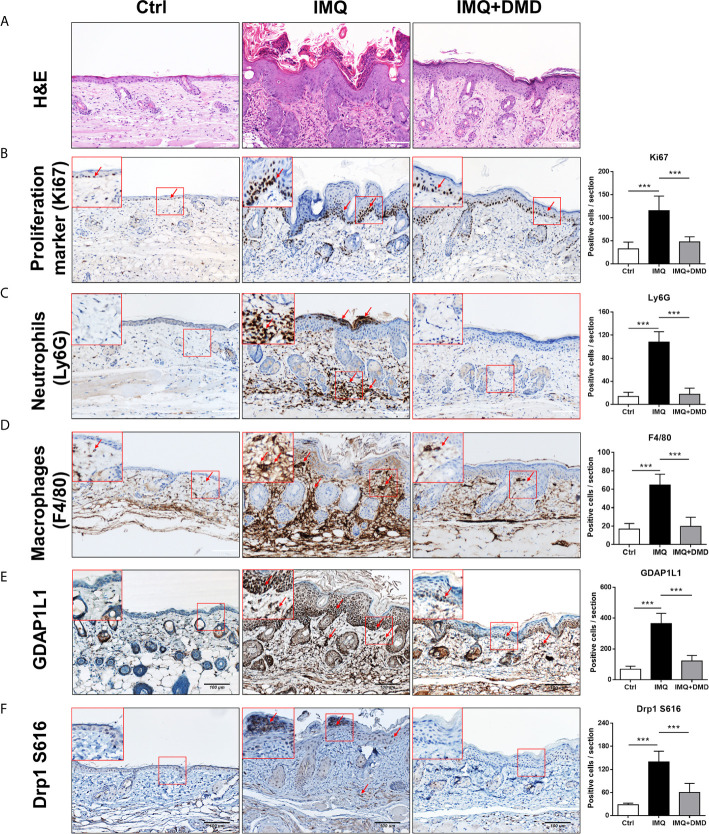
DMD attenuate IMQ-induced psoriasis-like inflammation in a mouse model as observed by skin histology. **(A)** Histological assessment of the skin by H&E staining. **(B)** Histological assessment of the skin by Ki67 staining. **(C)** Histological assessment of the skin by Ly6G staining. **(D)** Histological assessment of the skin by F4/80 staining. **(E)** Histological assessment of the skin by GDAP1L1 staining. **(F)** Histological assessment of the skin by Drp1 S616 staining. The upper left corner of each image indicates the higher power magnification of the red frame. The red arrow indicates the location of the antibody-stained cells. The quantification of the antibody-positive cells is shown in the right panel of each figure. ***P < 0.001 when compared to IMQ group. Data are represented as mean ± SEM. (n=9).

*In vivo* efficacy of DMD on psoriasiform lesions was qualitatively monitored using histology. Representative H&E staining from healthy skin revealed normal morphology with no damage to the epidermis and dermis (**Figure 5A**). H&E-stained IMQ-treated skin showed typical histological hallmarks of psoriasis, including acanthosis, hyperkeratosis, elongated rete ridge, and immune cell infiltration. IHC staining for Ki67, a proliferation biomarker revealed a remarkable increase of Ki67 in the basal layer after IMQ treatment (red arrows in **Figure 5B**). The higher power magnification of the histology is revealed in the upper left corner of each image. DMD effectively suppressed epidermal hyperproliferation in mice. The neutrophil infiltration in the stratum corneum and dermis could be visualized by Ly6G staining (red arrows in **Figure 5C**). The IHC showed clouds of Ly6G expression in the dermis after the IMQ challenge. In addition, DMD inhibited infiltrating neutrophils to a certain level. To detect macrophages, the skin was immunostained with F4/80 and its expression was observed in epidermal and dermal layers in IMQ-treated mouse (red arrows in **Figure 5D**), suggesting that macrophages were recruited to the lesional plaque. DMD could mitigate the macrophage infiltration. Strong immunoreactivity for GDAP1L1 and Drp1 S616 was observed in the psoriasiform lesion (red arrows in **Figures 5E, F**). Like the macrophage distribution, GDAP1L1 mainly expressed throughout the viable epidermis and dermis. Expression of both fission factors decreased after and DMD-treatment of the skin compared to IMQ stimulation alone. All quantification of the antibody-positive cell count was estimated by AlphaView software and depicted in the right panel of **Figure 5**. A statistically significant reduction of antibody-positive cell count was observed after topical administration of DMD on IMQ-treated mouse skin.

**Figure 6:**
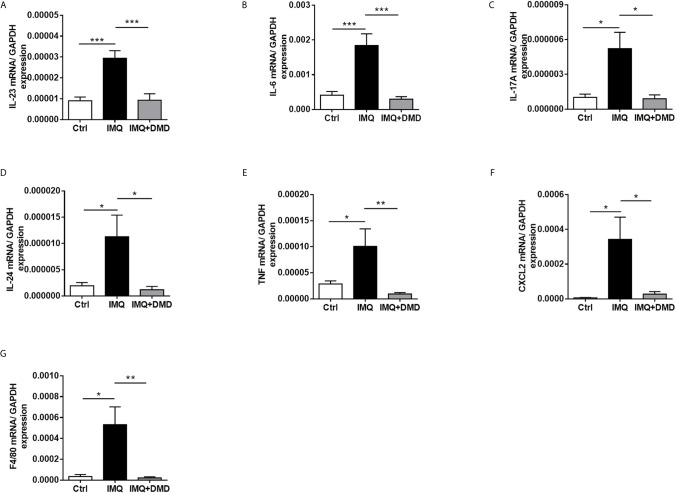
DMD suppress the expression of proinflammatory mediators in IMQ-induced psoriasiform lesion in mice. **(A–G)** The dorsal skin of mouse was topically treated with IMQ cream for 5 consecutive days. AC or DMD was also topically applied on the lesional skin if necessary. Total skin RNA was extracted and the mRNA level of IL-23, IL-6, IL-17A, IL-24, TNF, CXCL2, and F4/80 by RT-qPCR (*n*=8−12), respectively. *P < 0.05, **P < 0.01, ***P< 0.001 when compared to IMQ group. Data are represented as mean ± SEM. (*n*=6).

The mRNA level of the inflammation-related cytokines and chemokines including IL-23, IL-6, IL-17A, IL-24, TNF, and CXCL2 in mouse skin was determined through RT-qPCR and a dramatic increase in proinflammatory mediator expression was observed after the IMQ challenge (**Figures 6A–F**). The cytokines/chemokines in psoriasiform skin could be restrained to baseline control by DMD. These data clearly demonstrate the ability of DMD to reduce inflammation caused by IMQ. F4/80 mRNA level was significantly increased in IMQ-activated mouse skin and decreased after DMD application (**Figure 6G**). Collectively, the *in vivo* data prove the anti-inflammatory potential of DMD on the psoriasis-like lesions, especially on macrophages and IL-23/Th17 signaling.

## Discussion

Macrophages are thought to have a vital role in the induction of inflammation and autoimmune skin diseases such as psoriasis (1, 16). Therefore, we employed macrophages as the main cell model for evaluating the effect of DMD on the attenuation of psoriasis. The cutaneous macrophages in psoriatic plaque can release cytokines IL-6, TNF, and IL-1β for developing inflammation (22). Cytokines influence cell proliferation in psoriasis (23). Multiple cytokine-signaling including IL-23, IL-6, IL-24, and TNF is known to be important in psoriatic pathogenesis (24). The TLR stimulated by IMQ can initiate macrophage activation to express proinflammatory cytokines (25). Among these cytokines, IL-23 plays a preliminary role in macrophages to initiate psoriasis development (26). IL-23 is important for inducing IL-6-dependent epidermal hyperplasia and cytokine production in psoriasis. DMD could restrain the upregulation of IL-23 in the activated macrophages in the early stage, followed by the inhibition of IL-6 expression. In the psoriatic skin, one of the major sources of TNF is macrophage (4, 16). Upregulation of TNF was also largely inhibited by DMD. Psoriasis development can be triggered by TNF receptor 1-dependent upregulation of IL-24 (27). IL-24 is a member of the IL-20 family induced by IL-6, TNF, and IL-1β in psoriasis. Macrophages and T lymphocytes are the primary cells expressing IL-24 (28). Here, we confirmed the repression of IL-24 by DMD.

The cytokines including IL-24 and TNF-α mediate the crosstalk between immune cells and keratinocytes. The release of these cytokines stimulates keratinocyte proliferation and amplifies the inflammation in the psoriatic lesions (29). Macrophages accumulate in the psoriatic lesions and release cytokines to prompt the excessive proliferation of keratinocytes (30). We found that the conditioned medium of DMD-treated THP-1 could successfully inhibit keratinocyte proliferation and p-STAT3 expression. Hyperproliferation of keratinocytes in psoriasis correlates with the STAT3 pathway (31). STAT3 activation in keratinocytes is regarded as the main effector producing cytokines, which in turn leads to the positive feedback loop of psoriasis. DMD not only suppressed macrophage activation but also restrained the following interplay between macrophages and keratinocytes to restrict inflammation.

Another key factor generating the crosstalk between immune cells and keratinocytes is chemotaxis. Immune cell recruitment in psoriatic lesions depends on the chemotactic proteins, including chemokines and their receptors (32). The function of chemokines and cytokines shows some overlaps, but chemokines are primarily recognized for their capacity to modulate cell migration. Among the chemokines, CCL20 is a major contributor to immune cell infiltration in psoriasis (33). CCL20 expression is largely increased by TNF-α, IL-24, and IFN-γ (27, 29). CCL20 attracts dendritic cells and Th17 cells to sustain the inflammatory response through a feedback loop (26). DMD was found to significantly suppress CCL20, resulting in the blockage of the vicious inflammatory cycle. Besides CCL20, DMD was capable of inhibiting a series of chemokines and the receptors including CC, and CXC chemokines. CC chemokines majorly recruit T cells and monocytes, whereas CXC chemokines predominantly recruit neutrophils in psoriasis (34). For instance, enhanced CCL2 release by macrophages induces monocyte migration to the psoriasiform lesions in mice (35). CCL4L2 and CCL22 are pivotal chemokines in patients with psoriasis to stimulate the chemotactic infiltration of dendritic cells and macrophages (36, 37). A principal histopathological feature of psoriasis is the inflammatory infiltration of neutrophils in the stratum corneum (Munro’s microabscess) and dermis. Chemokine production by the stimulated macrophages acts as a chemoattractant for neutrophil accumulation (38). Our *in vitro* wound healing and transwell assays demonstrated the blockage of neutrophil migration after the suppression of chemokine subset in macrophages treated by DMD.

The binding of chemokines and some stimulators to the corresponding receptors can activate the downstream signaling pathways including those of MAPKs and NF-κB. IMQ is reported to bind with TLR, resulting in MAPKs phosphorylation and NF-κB to generate cytokines and chemokines (39). Overexpression of p-JNK, p-ERK, and p-p38 was inhibited by DMD, suggesting the possible role of this compound in MAPK regulation. Among MAPKs, ERK regulates IL-24 expression in psoriatic lesions (40). Our result verified IL-24 downregulation by DMD through the ERK-dependent signaling. MAPKs regulate the downstream signaling of the transcription factor NF-κB in macrophages to produce cytokines and chemokines for psoriasis development (41). We speculated that both MAPKs and NF-κB pathways participate in DMD-induced cytokine/chemokine arrest.

The RNA sequencing study showed a potential role of GDAP1L1 in the anti-inflammatory activity of DMD against macrophages. GDAP1L1 is a mitochondrial fission factor governing the mitochondrial dynamics. Under oxidative stress, GDAP1L1 translocates to the mitochondria. Mitochondrial fission and fusion need to be controlled to keep the balance required for the persistence of mitochondrial morphology. Mitochondrial dynamics mediate energy output, quality control, and the generation of reactive oxygen species (ROS) (42). The defect in mitochondrial membrane dynamics predominantly affects neuron functions to cause neurodegenerative diseases (43). Mitochondrial fission raised as a result of a high level of stress may be one of the main causes of inflammation and immune dysregulation (44). Due to its central role in mitochondrial fission, Drp1 is a prime target for regulatory pathways. Drp1 mainly exists in the cytosol but partially moves to the outer membrane of mitochondria for mediating the process of fission (45). Drp1-mediated fragmentation leads to ROS generation, which is implicated in the pathogenesis of Alzheimer’s and Parkinson’s diseases (46). Translocation of Drp1 to mitochondria depends on the phosphorylation at S616. Phosphorylation regulates the cycling of Drp1 between the cytoplasm and mitochondrial membrane to prompt fission (47). In this study, IMQ treatment-induced GDAP1L1 expression and translocation, which is involved in the proinflammatory factor expression. GDAP1L1 could induce inflammation in the presence of Drp1 and inhibition of GDAP1L1 and Drp1 S616 translocation to mitochondria mediated by DMD prevented macrophage activation. Some fission factors are associated with apoptotic induction, whereas GDAP1L1 elicits mitochondrial fragmentation without inducing apoptosis (48). Thus, we ruled out the apoptosis pathway mediating the effect of DMD on macrophage activation.

Mitochondrial fission may be fundamental for dominating the production of proinflammatory mediators. A previous study (49) demonstrated that downregulation of Drp1 attenuates proinflammatory factors *via* the reduced MAPK and NF-κB signaling in microglial cells. ERK can trigger mitochondrial fission through phosphorylation of Drp1 at S616 and its translocation to mitochondria (50). Our experimental data inferred that DMD arrested MAPK signaling, blocking the translocation of Drp1 S616 and the subsequent fission. The mechanisms by which GDAP1L1/Drp1 axis regulates macrophage activation are unclear. There is little evidence to link GDAP1L1/Drp1 with inflammation. Zhang et al. (51) reported that Drp1 gene expression increases in atopic dermatitis, and other autoimmune skin diseases. However, Therianou et al. (52) demonstrated a contrary result in lesional psoriatic skin. Further study is needed to yield detailed insight into the molecular pathways that mediate GDAP1L1/Drp1 translocation in psoriasis.

The topical treatment of IMQ cream on murine skin creates inflammation resembling the symptoms of human psoriasis (20). IMQ caused phenotypic changes in psoriasis, including epidermal hyperplasia, inflammatory cell infiltration, and IL-23/Th17 axis activation. Relieving these pathological events is critical for psoriasis management. DMD effectively ameliorated IMQ-triggered inflammation and hyperproliferation. The epidermal thickness of IMQ-treated mouse skin could be reduced by 47% after DMD application. Based on the same protocol of psoriasis-like lesion induction by IMQ, our previous data (53) demonstrated that betamethasone as a positive control could decrease epidermal thickness by 43%. This indicates a comparable therapeutic efficacy between DMD and the drug used in clinics. IHC and RT-qPCR results manifested a large accumulation of macrophages and neutrophils in viable skin of the psoriasiform lesion. This observation confirmed the macrophage recruitment in the epidermis and dermis of IMQ-treated mouse skin as was proved in the previous study (54). Our experimental data convincingly demonstrated that a decrease in macrophage recruitment by topical DMD contributed to the resolution of psoriasiform inflammation. We found that DMD application inhibited IMQ-induced overexpression of GDAP1L1 and Drp1 in mouse skin. IL-23 and IL-17 are highly involved in the IMQ-induced animal model of psoriasis (55). In psoriatic lesions, IL-23 is greatly expressed in dendritic cells and macrophages (56). IL-17A is important in relieving psoriasis by upregulating cytokines IL-6, IL-1β, and TNF-α (57). Overexpressed IL-17A in psoriatic skin leads to the proliferation and abnormal differentiation of keratinocytes (58). Macrophages express low levels of IL-17A (16). Psoriasis is a complex disease with a dynamic interaction between immune cells and keratinocytes, as well as their cytokines and chemokines (59). Activation of a confederacy of cell types in psoriasis includes T cells, mast cells, dendritic cells, neutrophils, macrophages, and keratinocytes. As psoriasis develops, T cells and neutrophils are reported to release IL-17A (16, 60). Our data manifested downregulation of IL-17A in IMQ-treated skin by DMD, which might directly or indirectly act on different immune cells with or without macrophage intervention. IL-17A further recruits dendritic cells and Th17 cells to the psoriatic lesion. The production of IL-1β and TNF-α from macrophages can be stimulated by IL-17A (61), which also acts on keratinocytes to increase their proliferation and chemokine expression. Blocking IL-17A by DMD led to the inhibition of epidermal thickening as detected in our study. IL-24 is another cytokine largely produced by macrophages to direct keratinocyte proliferation (33). In addition to macrophages, IL-24 is produced by T cells, mast cells, and keratinocytes in psoriatic plaque (40). The inhibition of IL-24 by topically applied DMD could be beneficial in diminishing keratinocyte proliferation according to IHC. Neutralization of cytokines by DMD not only decreased the number of macrophages in viable skin, but also the number of neutrophils in the stratum corneum and dermis. Inhibition of neutrophil recruitment impeded the aberrant interplay between neutrophils and keratinocytes, thus blocking the keratinocyte hyperproliferation induced by neutrophil cytokines/chemokines. The significant reduction of GDAP1L1 and Drp1 S616 in IMQ-stimulated skin by DMD corroborated with the *in vitro* result of translocation inhibition.

**Figure 7:**
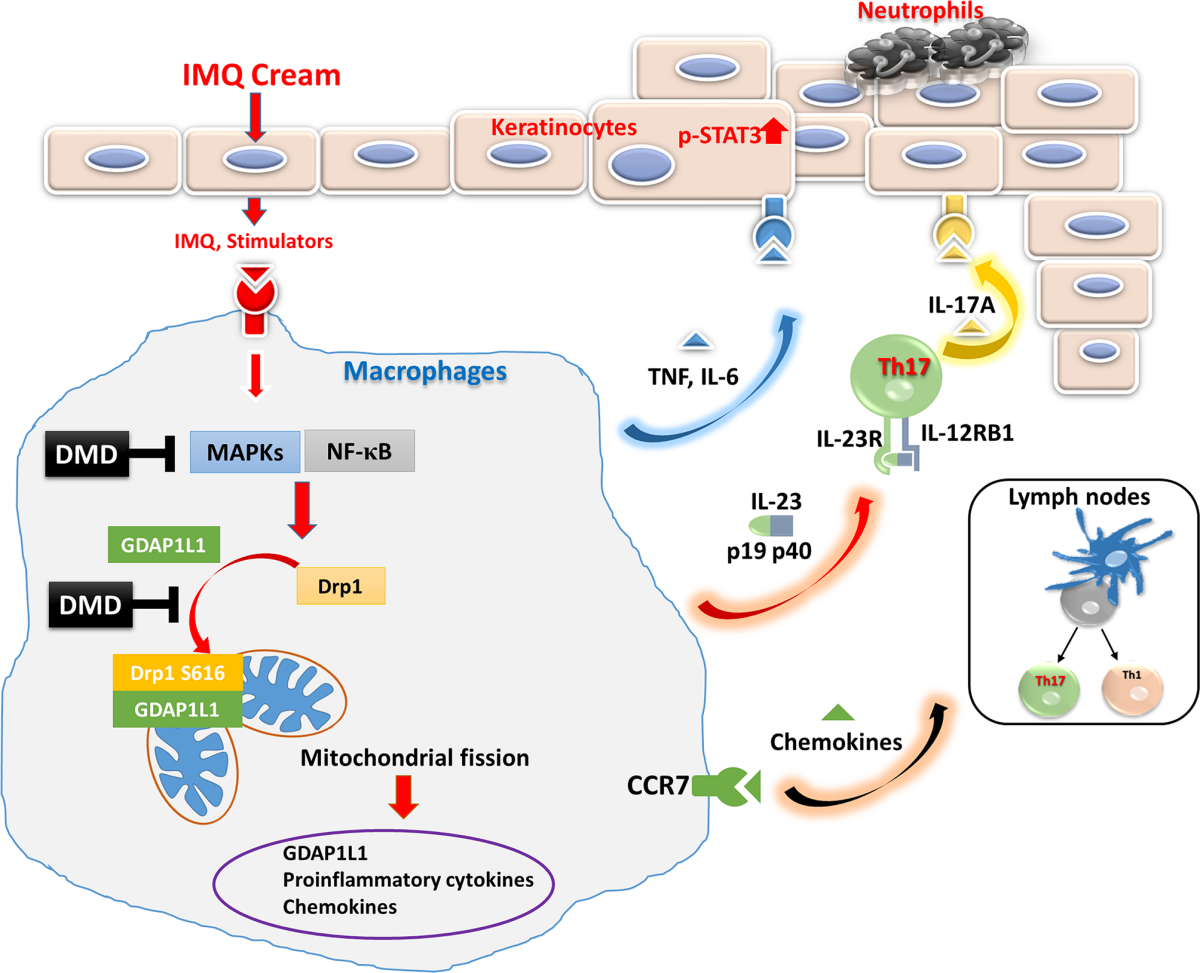
Schematic representation of the proposed mechanisms responsible for the anti-inflammatory effect of DMD in macrophages. We found that IMQ stimulation induced GDAP1L1 expression and GDAP1L1-dependent Drp1 S616 phosphorylation, and then caused mitochondria translocation of GDAP1L1 and phosphorylated Drp1 S616, leading to mitochondrial fission. IMQ stimulation also induced inflammatory mediators’ expression as a result of the activation of NF-κB and MAPK phosphorylation, which was the GDAP1L1-dependent manner. We demonstrate that DMD prevented IMQ-induced mitochondrial fission by blocking GDAP1L1-dependent Drp1 S616 phosphorylation and mitochondria translocation. It could suppress proinflammatory mediator expression in activated macrophages. DMD treatment suppressed migration of activated macrophages into the inflamed site of the mouse skin. The cytokines and chemokines from macrophages in turn rescue neutrophils and cause epidermal proliferation were blocked by DMD treatment.

The findings in the mechanistic and animal studies suggest the multiple antipsoriatic mechanisms of DMD. This compound suppressed IMQ-induced expression of proinflammatory cytokines/chemokines by obstructing of MAPK and NF-κB phosphorylation in macrophages. We also verified that DMD downregulated proinflammatory factors *via* the inhibition of GDAP1L1 and Drp1 translocation and Drp1 phosphorylation. This inhibition might be mediated by decreasing the overexpression of p-NF-κB. Further neutrophil recruitment and keratinocyte hyperproliferation could be prevented by inhibiting macrophage activation after DMD management. The possible mechanisms of action of DMD are depicted in **Figure 7**. The safety of topically applied DMD was examined earlier and shows a negligible irritation in healthy mouse skin (14). A satisfactory therapeutic efficacy and safety could be achieved for topical DMD application.

## Conclusions

The present work investigated the anti-inflammatory potential of a pure compound (DMD) derived from *A. cinnamomea* for mitigating psoriasiform plaque. The *in vitro* assay on THP-1 cells presented DMD-mediated inhibition of overexpressed cytokine/chemokine. The conditioned medium of the activated macrophages treated with DMD could suppress neutrophil migration and keratinocyte proliferation. The *in vivo* animal study showed relief in psoriasiform symptoms after DMD treatment. In addition, cytokine/chemokine upregulation and macrophage recruitment in the psoriasis-like lesion were alleviated *in vivo*. DMD downregulated proinflammatory mediators through the MAPKs and NF-κB pathways. The finding in this study also revealed that the GDAP1L1/Drp1 signaling axis plays a critical role in macrophages as an activator of IMQ-induced proinflammatory factors. This axis could be a target of anti-inflammatory DMD to reduce the lesions. This natural compound, DMD, sheds new light on the therapeutic approach against psoriasis.

## Data Availability Statement

The datasets of transcriptomic analysis for this study can be found on the website Gene Expression Omnibus (https://www.ncbi.nlm.nih.gov/geo/). The accession number is GSE171667.

## Ethics Statement

The studies involving human participants were reviewed and approved by Institutional Review Board at Chang Gung Memorial Hospital. The patients/participants provided their written informed consent to participate in this study.

## Author Contributions

S-YC initiated the study and drafted the manuscript. C-YC involved in the design of all experiments. S-YC and S-CY carried out the experiments. S-YC and AA analyzed data and wrote the manuscript. C-HL and J-YF supervised the entire project. J-YF reviewed critically and approved the final manuscript. All authors contributed to the article and approved the submitted version.

## Conflict of Interest

The authors declare that the research was conducted in the absence of any commercial or financial relationships that could be construed as a potential conflict of interest.
