# Supplementary material for: 2,4-Dimethoxy-6-Methylbenzene-1,3-diol, a Benzenoid From Antrodia cinnamomea, Mitigates Psoriasiform Inflammation by Suppressing MAPK/NF-κB Phosphorylation and GDAP1L1/Drp1 Translocation
Source: Front Immunol. 2021 May 14;12:664425. doi: 10.3389/fimmu.2021.664425 (PMC8162112; doi:10.3389/fimmu.2021.664425)
Supplement: Supplementary file 1 [file Presentation_1.pptx]

## Slide 1
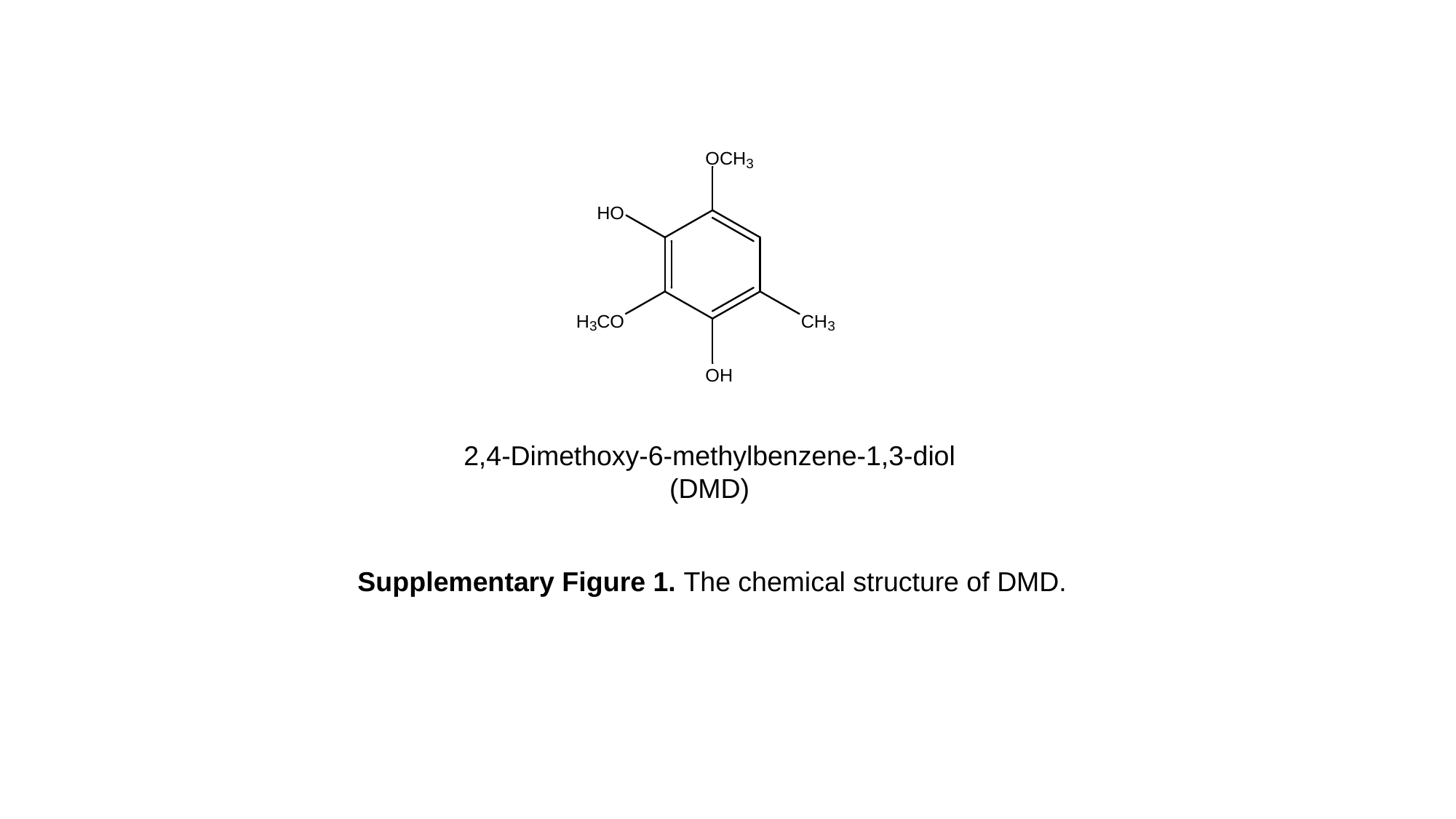

2,4-Dimethoxy-6-methylbenzene-1,3-diol (DMD)
Supplementary Figure 1. The chemical structure of DMD.

## Slide 2
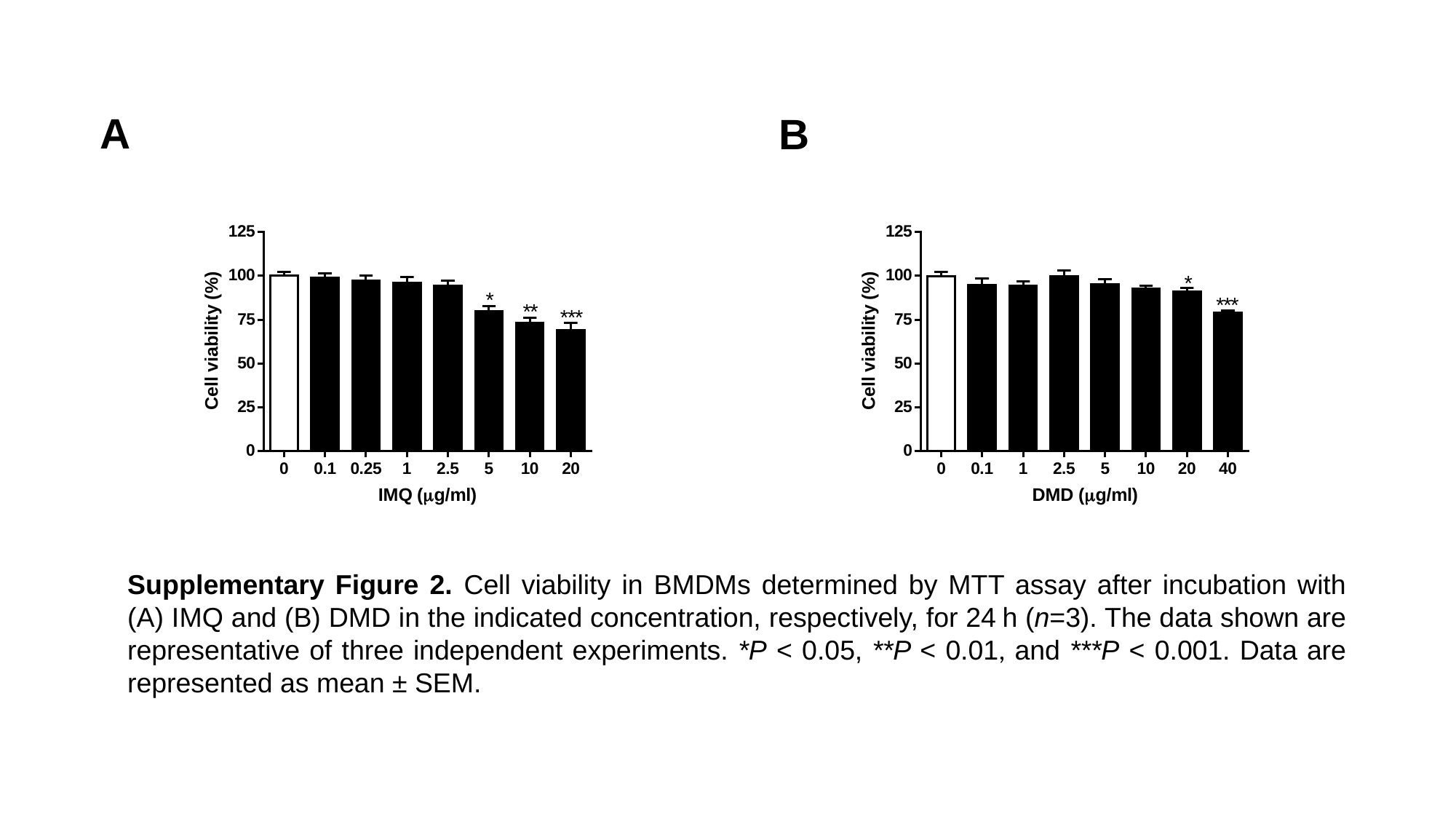

A
B
Supplementary Figure 2. Cell viability in BMDMs determined by MTT assay after incubation with (A) IMQ and (B) DMD in the indicated concentration, respectively, for 24 h (n=3). The data shown are representative of three independent experiments. *P < 0.05, **P < 0.01, and ***P < 0.001. Data are represented as mean ± SEM.

## Slide 3
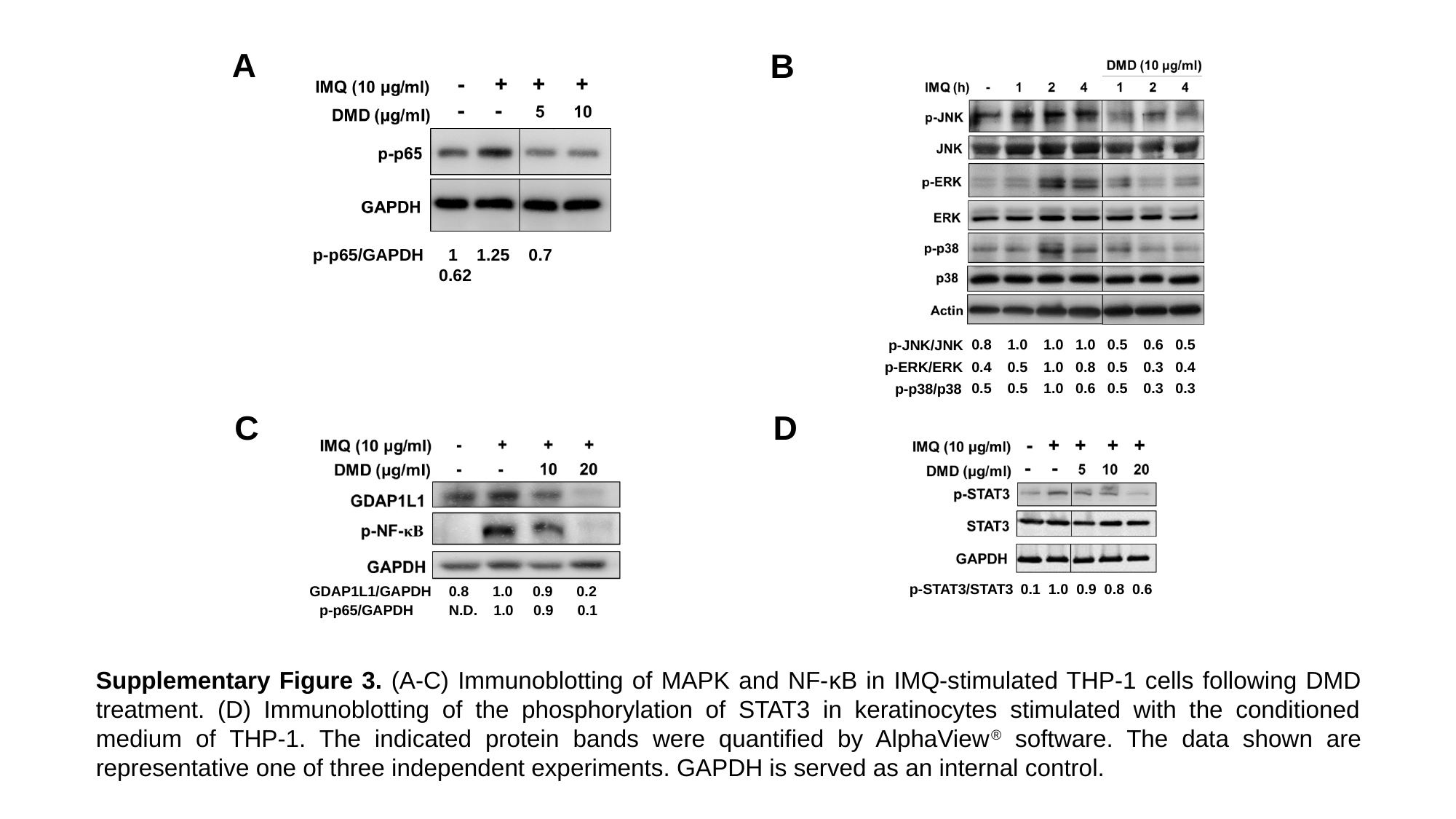

A
B
0.8 1.0 1.0 1.0 0.5 0.6 0.5
p-JNK/JNK
p-ERK/ERK
0.4 0.5 1.0 0.8 0.5 0.3 0.4
0.5 0.5 1.0 0.6 0.5 0.3 0.3
p-p38/p38
p-p65/GAPDH
 1 1.25 0.7 0.62
C
D
p-STAT3/STAT3
0.1 1.0 0.9 0.8 0.6
GDAP1L1/GAPDH
0.8 1.0 0.9 0.2
p-p65/GAPDH
N.D. 1.0 0.9 0.1
Supplementary Figure 3. (A-C) Immunoblotting of MAPK and NF-κB in IMQ-stimulated THP-1 cells following DMD treatment. (D) Immunoblotting of the phosphorylation of STAT3 in keratinocytes stimulated with the conditioned medium of THP-1. The indicated protein bands were quantified by AlphaView® software. The data shown are representative one of three independent experiments. GAPDH is served as an internal control.

## Slide 4
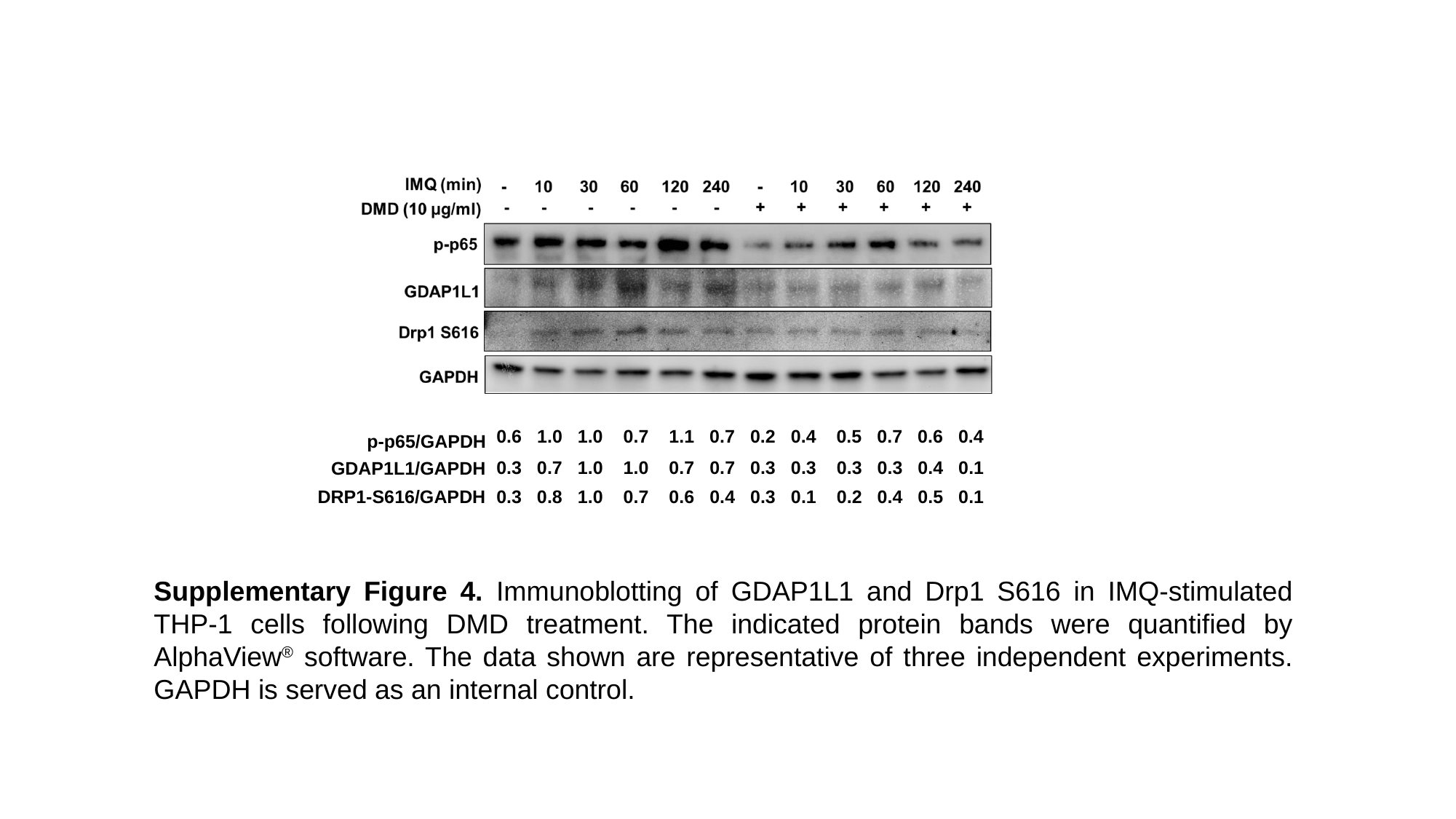

0.6 1.0 1.0 0.7 1.1 0.7 0.2 0.4 0.5 0.7 0.6 0.4
p-p65/GAPDH
0.3 0.7 1.0 1.0 0.7 0.7 0.3 0.3 0.3 0.3 0.4 0.1
GDAP1L1/GAPDH
DRP1-S616/GAPDH
0.3 0.8 1.0 0.7 0.6 0.4 0.3 0.1 0.2 0.4 0.5 0.1
Supplementary Figure 4. Immunoblotting of GDAP1L1 and Drp1 S616 in IMQ-stimulated THP-1 cells following DMD treatment. The indicated protein bands were quantified by AlphaView® software. The data shown are representative of three independent experiments. GAPDH is served as an internal control.

## Slide 5
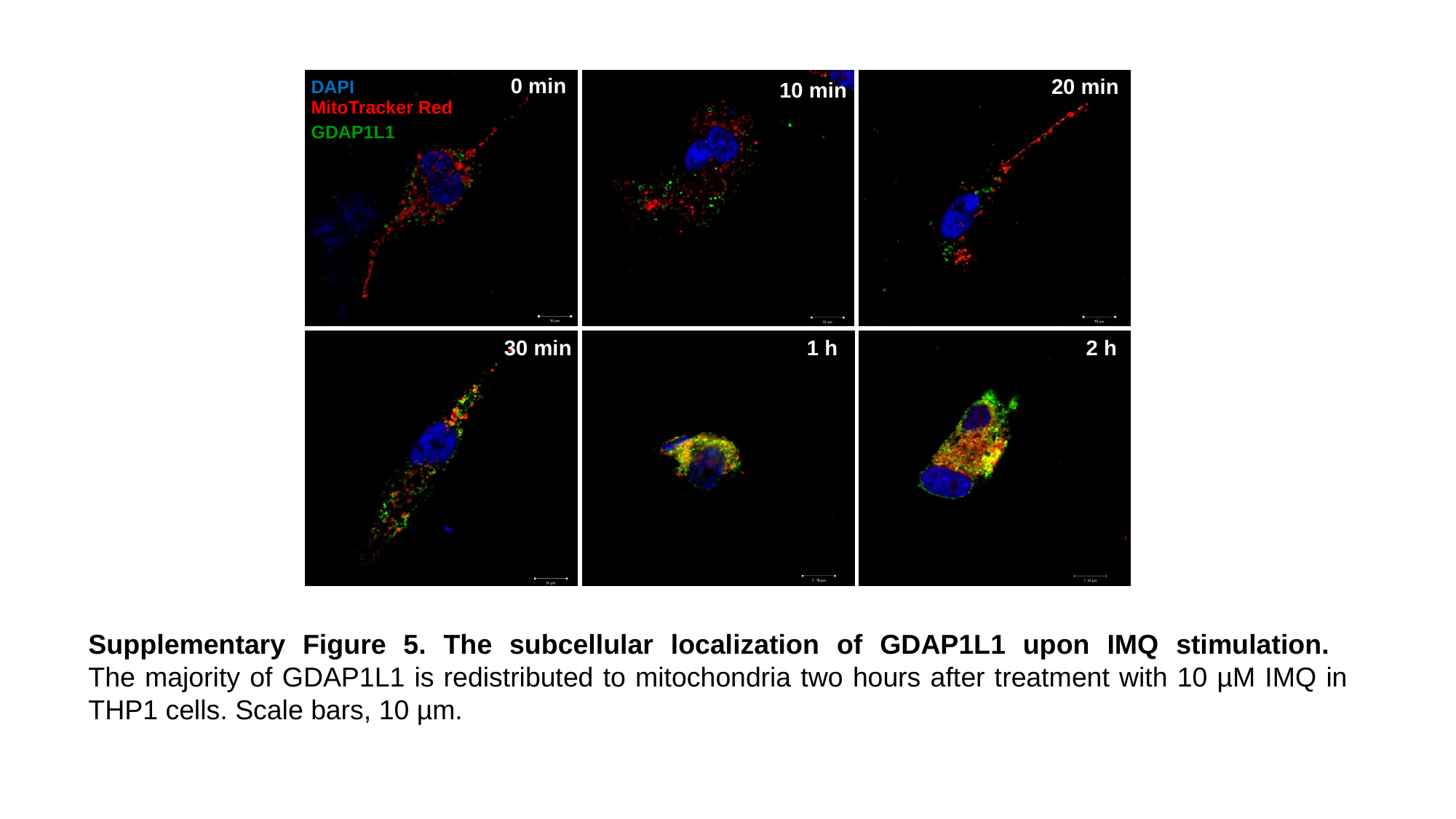

0 min
20 min
10 min
30 min
1 h
2 h
DAPI
MitoTracker Red
GDAP1L1
Supplementary Figure 5. The subcellular localization of GDAP1L1 upon IMQ stimulation.
The majority of GDAP1L1 is redistributed to mitochondria two hours after treatment with 10 µM IMQ in THP1 cells. Scale bars, 10 µm.
