# Supplementary material for: 2,4-Dimethoxy-6-Methylbenzene-1,3-diol, a Benzenoid From Antrodia cinnamomea, Mitigates Psoriasiform Inflammation by Suppressing MAPK/NF-κB Phosphorylation and GDAP1L1/Drp1 Translocation
Source: Front Immunol. 2021 May 14;12:664425. doi: 10.3389/fimmu.2021.664425 (PMC8162112; doi:10.3389/fimmu.2021.664425)
Supplement: Supplementary file 2 [file Presentation_2.pptx]

## Slide 1
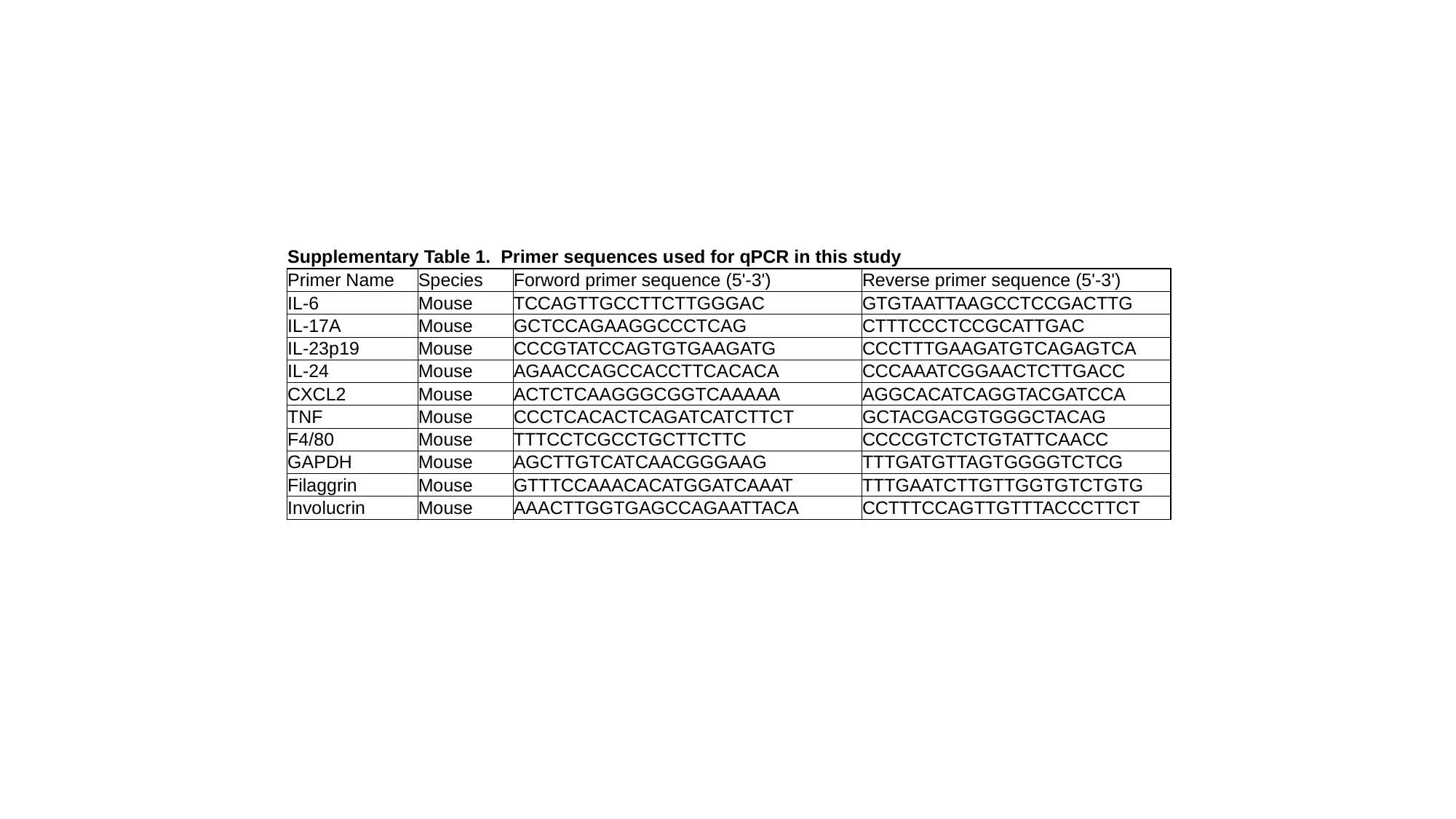

| Supplementary Table 1. Primer sequences used for qPCR in this study | | | |
| --- | --- | --- | --- |
| Primer Name | Species | Forword primer sequence (5'-3') | Reverse primer sequence (5'-3') |
| IL-6 | Mouse | TCCAGTTGCCTTCTTGGGAC | GTGTAATTAAGCCTCCGACTTG |
| IL-17A | Mouse | GCTCCAGAAGGCCCTCAG | CTTTCCCTCCGCATTGAC |
| IL-23p19 | Mouse | CCCGTATCCAGTGTGAAGATG | CCCTTTGAAGATGTCAGAGTCA |
| IL-24 | Mouse | AGAACCAGCCACCTTCACACA | CCCAAATCGGAACTCTTGACC |
| CXCL2 | Mouse | ACTCTCAAGGGCGGTCAAAAA | AGGCACATCAGGTACGATCCA |
| TNF | Mouse | CCCTCACACTCAGATCATCTTCT | GCTACGACGTGGGCTACAG |
| F4/80 | Mouse | TTTCCTCGCCTGCTTCTTC | CCCCGTCTCTGTATTCAACC |
| GAPDH | Mouse | AGCTTGTCATCAACGGGAAG | TTTGATGTTAGTGGGGTCTCG |
| Filaggrin | Mouse | GTTTCCAAACACATGGATCAAAT | TTTGAATCTTGTTGGTGTCTGTG |
| Involucrin | Mouse | AAACTTGGTGAGCCAGAATTACA | CCTTTCCAGTTGTTTACCCTTCT |
