# Supplementary material for: 2,4-Dimethoxy-6-Methylbenzene-1,3-diol, a Benzenoid From Antrodia cinnamomea, Mitigates Psoriasiform Inflammation by Suppressing MAPK/NF-κB Phosphorylation and GDAP1L1/Drp1 Translocation
Source: Front Immunol. 2021 May 14;12:664425. doi: 10.3389/fimmu.2021.664425 (PMC8162112; doi:10.3389/fimmu.2021.664425)
Supplement: Supplementary file 3 [file Presentation_3.pptx]

## Slide 1
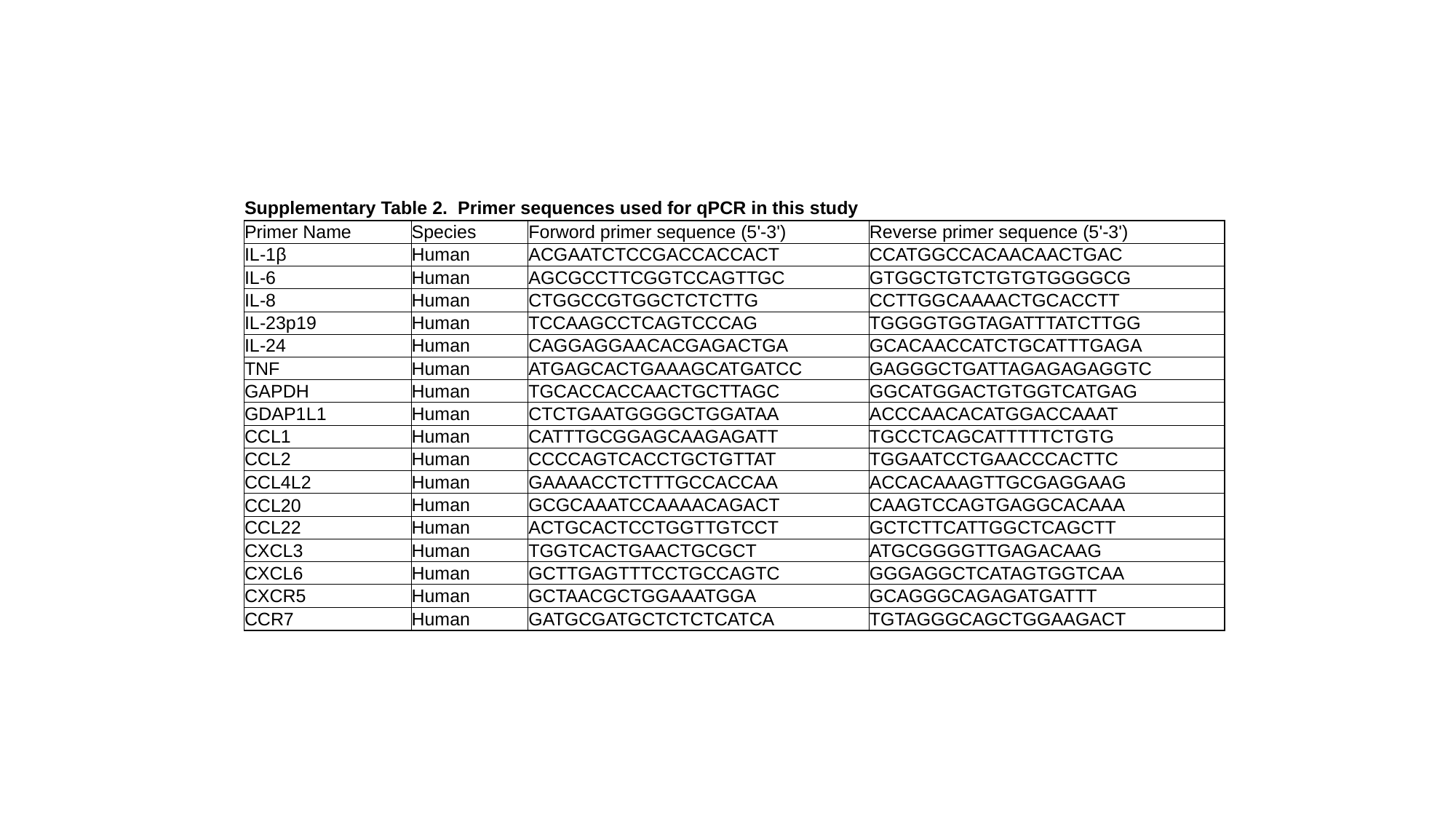

| Supplementary Table 2. Primer sequences used for qPCR in this study | | | |
| --- | --- | --- | --- |
| Primer Name | Species | Forword primer sequence (5'-3') | Reverse primer sequence (5'-3') |
| IL-1β | Human | ACGAATCTCCGACCACCACT | CCATGGCCACAACAACTGAC |
| IL-6 | Human | AGCGCCTTCGGTCCAGTTGC | GTGGCTGTCTGTGTGGGGCG |
| IL-8 | Human | CTGGCCGTGGCTCTCTTG | CCTTGGCAAAACTGCACCTT |
| IL-23p19 | Human | TCCAAGCCTCAGTCCCAG | TGGGGTGGTAGATTTATCTTGG |
| IL-24 | Human | CAGGAGGAACACGAGACTGA | GCACAACCATCTGCATTTGAGA |
| TNF | Human | ATGAGCACTGAAAGCATGATCC | GAGGGCTGATTAGAGAGAGGTC |
| GAPDH | Human | TGCACCACCAACTGCTTAGC | GGCATGGACTGTGGTCATGAG |
| GDAP1L1 | Human | CTCTGAATGGGGCTGGATAA | ACCCAACACATGGACCAAAT |
| CCL1 | Human | CATTTGCGGAGCAAGAGATT | TGCCTCAGCATTTTTCTGTG |
| CCL2 | Human | CCCCAGTCACCTGCTGTTAT | TGGAATCCTGAACCCACTTC |
| CCL4L2 | Human | GAAAACCTCTTTGCCACCAA | ACCACAAAGTTGCGAGGAAG |
| CCL20 | Human | GCGCAAATCCAAAACAGACT | CAAGTCCAGTGAGGCACAAA |
| CCL22 | Human | ACTGCACTCCTGGTTGTCCT | GCTCTTCATTGGCTCAGCTT |
| CXCL3 | Human | TGGTCACTGAACTGCGCT | ATGCGGGGTTGAGACAAG |
| CXCL6 | Human | GCTTGAGTTTCCTGCCAGTC | GGGAGGCTCATAGTGGTCAA |
| CXCR5 | Human | GCTAACGCTGGAAATGGA | GCAGGGCAGAGATGATTT |
| CCR7 | Human | GATGCGATGCTCTCTCATCA | TGTAGGGCAGCTGGAAGACT |
